# Inevitable high-dose irradiation to lead of implantable cardioverter defibrillator in small cell lung cancer: a case report

**DOI:** 10.1186/s13256-019-2111-y

**Published:** 2019-06-20

**Authors:** Jeong Won Lee, Ki Ho Seol

**Affiliations:** Department of Radiation Oncology, Catholic University of Daegu School of Medicine, 33, Duryugongwon-ro 17-gil, Nam-gu, Daegu, South Korea

**Keywords:** Implantable cardioverter-defibrillator, Lung cancer, Radiotherapy

## Abstract

**Background:**

Radiotherapy has been shown to cause malfunction of implantable cardioverter-defibrillators, and there are few studies of implantable cardioverter-defibrillators and radiotherapy. We report an unusual case of small cell lung cancer in a patient with an implantable cardioverter-defibrillator in whom direct irradiation to the electrode and lead could not be avoided.

**Case presentation:**

We report a case of radiotherapy in a 72-year-old Korean man with a limited stage of small cell lung cancer who had undergone insertion of an implantable cardioverter-defibrillator because of ventricular fibrillation. The radiation dose was 60 Gy in 30 fractions to the thorax. The mean dose and maximum dose estimated at the body of the implantable cardioverter-defibrillator were 0.89 Gy and 2.23 Gy, respectively. The mean and maximum doses of the lead and electrode were 17.12 Gy and 55.72 Gy in the lead and 1.81 Gy and 7.10 Gy in the electrode, respectively, because part of the lead and electrode was inevitably in the irradiated fields. The function of the patient’s implantable cardioverter-defibrillator was checked daily, and no change in implantable cardioverter-defibrillator function was observed for the duration of radiotherapy. The patient was tolerated the treatment well without severe complications. Computed tomography performed at 4 weeks after radiotherapy showed a good response with regression of the tumor. The patient was alive with complete remission of the tumor and without any implantable cardioverter-defibrillator dysfunction more than 36 months after the end of treatment.

**Conclusions:**

This case demonstrates that radiotherapy may be a safe and effective treatment modality through careful monitoring of implantable cardioverter-defibrillators in patients with lung cancer who have implantable cardioverter-defibrillators.

## Background

As the use of cardiac implantable electronic devices (CIEDs) such as permanent pacemakers or implantable cardioverter-defibrillators (ICDs) in the management of cardiovascular disease has increased with increasing life expectancy, so has the indication of radiotherapy in comorbidity of cancer and cardiovascular disease with CIEDs [1]. Radiotherapy has been shown to cause malfunction of CIEDs, ranging from device programming, to inappropriate triggering or inhibition of device therapies, or to complete device failure [2–4]. There are a few studies of ICD and radiotherapy, citing values of 1–2 Gy for a tolerable cumulative radiotherapy dose, which is an estimate requiring further research [2–5]. Although ICDs are composed of a body (generator) and wires (electrode and lead), these reports were chiefly focused on the pacemaker or the body of the ICD. The effect of radiotherapy on electrodes and leads of ICDs are unclear. We present an unusual case of small cell lung cancer in a patient with an ICD who could not avoid direct irradiation to the electrode and lead, and we describe his successful radiotherapy outcome.

## Case presentation

**Fig. 1:**
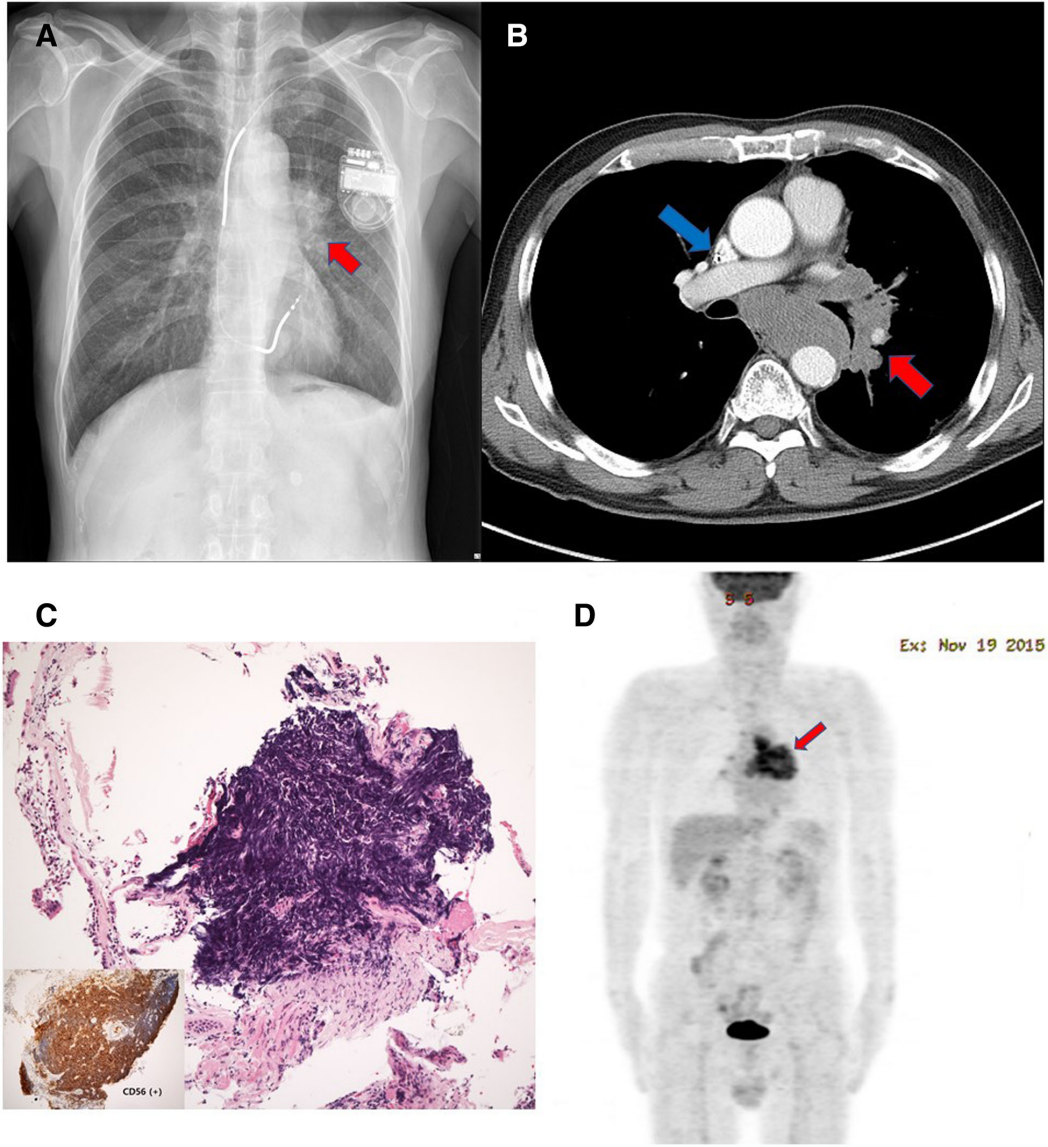
Disease presentation. **a** Simple chest radiography (red arrow: lung mass). **b** Computed tomography (red arrow: lung mass, blue arrow: lead of implantable cardioverter-defibrillators). **c** Photomicrograph of bronchoscopic biopsy. **d** Positron emission tomography (red arrow: lung mass)

A 72-year-old Korean man with a past medical history of ICD insertion for idiopathic ventricular fibrillation (device: Medtronic Protecta XT VRD354VRM; lead: Medtronic Sprint Quattro Secure Model 6947) presented with a 1-month history of complaint of a dry cough. He had a 50–pack-year history of smoking. His family history was negative for any malignancy. Chest x-ray and contrast-enhanced computed tomography showed a conglomerate nodal mass in the left central lung and left hilar area (Fig. 1a, b). Bronchoscopy was performed, and the cell block obtained from a needle biopsy was evaluated. A photomicrograph of the bronchoscopic biopsy showed a nest of atypical cells that squeezed hyperchromatic nuclei (Fig. 1c). IHC showed that these cells were positive for neuroendocrine markers, such as CD56 and chromogranin, and negative for CD45RO. The patient had an elevated serum lactate dehydrogenase level (337 U/L). Positron emission tomography excluded any additional disease localizations (Fig. 1d). The patient was diagnosed with a limited stage of small cell lung cancer in the left lung (cT4N2M0 by TNM staging).

The patient was recommended for concurrent chemoradiotherapy (CCRT), but he refused CCRT because of fear of toxicity. The tumor showed partial remission after four cycles of chemotherapy (cisplatin 25 mg/m^2^ on days 1, 2, and 3 and etoposide 100 mg/m^2^ on days 1, 2, and 3). He was referred for sequential thoracic radiotherapy. After a multidisciplinary meeting, we decided to treat him with radiotherapy and that the condition of his ICD would be monitored by a cardiologist during radiotherapy.

**Fig. 2:**
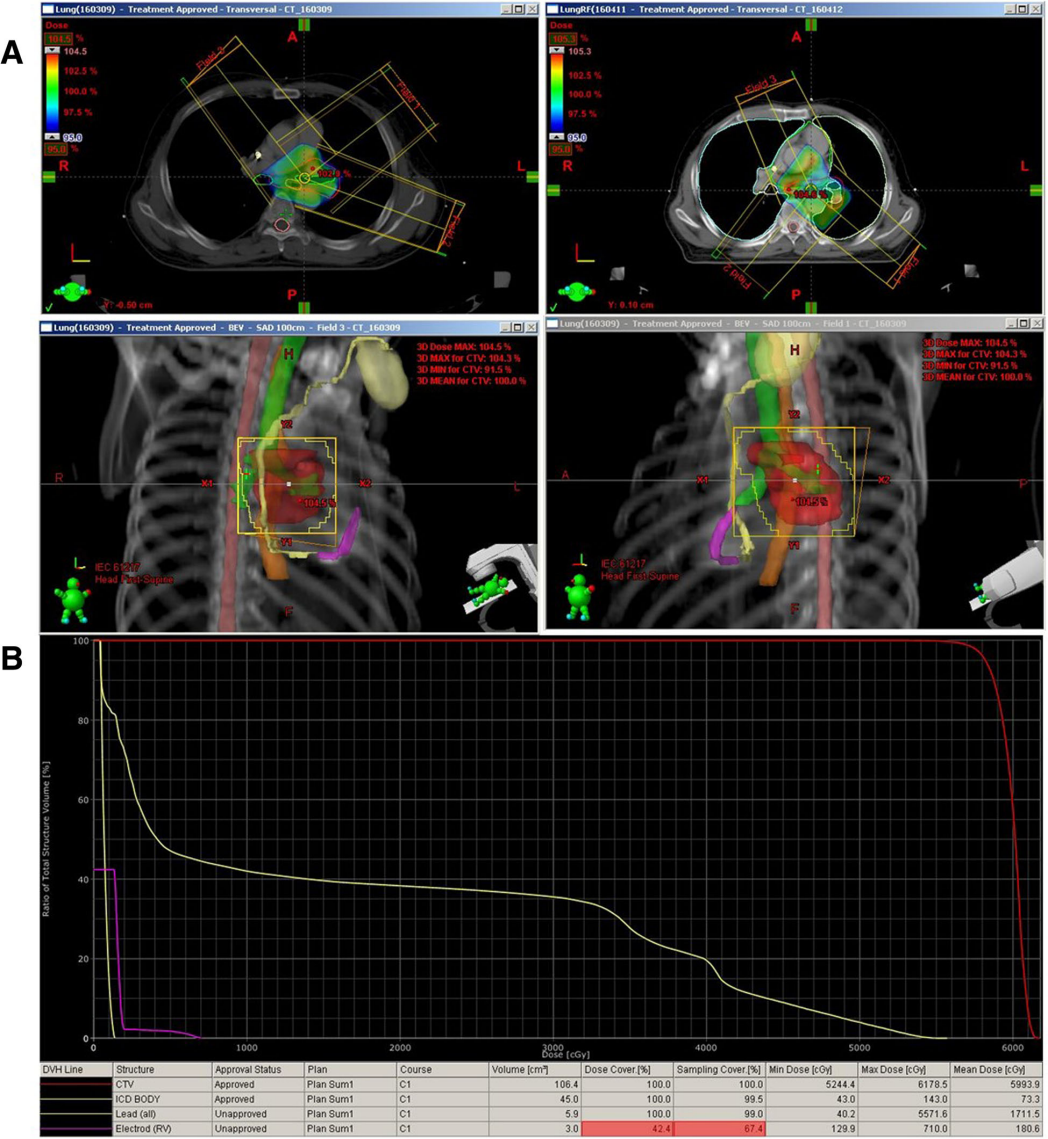
Radiotherapy planning. **a** Treatment field arrangement of radiotherapy. **b** Dose-volume histogram of planned radiotherapy

The primary tumor, regional gross lymph nodes, and surrounding normal structures were contoured in radiotherapy planning computed tomography. For ICD delineation, three parts of the ICD were contoured: the body of the ICD, the leads, and the electrode. Dose calculation was performed using analytical anisotropic algorithm (version 8.9.17). The goal of treatment planning was to achieve a dose to the target volume greater than 97% of the prescribed dose while minimizing the dose to surrounding normal organs and avoiding the directly irradiated field of beam arrangement into the contour of the lead and electrode. The prescribed dose was 60 Gy in 30 fractions five times per week. On dose-volume histogram (DVH) analysis, the mean and maximum doses of ICD were 0.73 Gy and 1.43 Gy, respectively, in the body. The mean and maximum doses of the lead and electrode were 17.12 Gy and 55.72 Gy in the lead and 1.81 Gy and 7.10 Gy in the electrode, respectively; this was because parts of the lead and electrode were inevitably in the irradiation fields. Radiation was delivered by linear accelerator (Varian Clinac 21EX; Varian Medical Systems, Palo Alto, CA, USA) using 10-MV 3-fixed photon beams in two sequential dosimetric treatment planning steps (Fig. 2).

Prior to each treatment, we placed a magnet on the ICD to suspend tachyarrhythmia detection because this can prevent ICD failure, and we used electronic portal imaging for image guidance. The ICD dose was calculated using a metal oxide semiconductor field effect transistor (MOSFET) dosimeter above an external magnet superposed on the ICD during every treatment. The mean and maximum doses estimated at the body of the ICD *in vivo* were 0.89 Gy and 2.23 Gy, respectively.

The function of the patient’s ICD was checked daily, and no change was observed during radiotherapy. The patient showed good tolerance without severe complications. Computed tomography performed at 4 weeks after radiotherapy showed good response with tumor regression. The patient remained in complete remission without ICD dysfunction more than 36 months after treatment completion.

## Discussion and conclusions

The use of ICDs in management of cardiovascular disease has increased with increasing life expectancy and the aging population. The improvements of ICD have prolonged survival in patients with previous arrhythmia. This has increased the morbidity of malignant disease in patients with cardiac devices. Cancer therapy in patients with cardiac devices is limited because of the problem of cardiac function or concurrent medical comorbidities. Surgery and systemic therapy, such as chemotherapy, may be unsuitable for these patients. Radiotherapy may be the best way to treat malignant disease. In this case, there are many concerns regarding radiotherapy for patients with ICD, including the “safe dose” for devices, the kinds of errors that may occur in the devices, and the care required for patients and devices during radiotherapy.

Radiotherapy has been shown to cause malfunction of CIEDs, ranging from device programming, to inappropriate triggering or inhibition of device therapies, or to complete device failure [1–4]. Radiotherapy-induced CIED failure was reported to be 2.5% in pacemakers and 6.8% in ICDs [1]. According to Hurkmans *et al.*, an ICD is likely to be more responsive than a pacemaker to radiation [5]. Therefore, it is necessary to be careful when planning and delivering radiation to patients with ICD. In 2015, Zaremba *et al*. summarized the checkpoints of CIEDs for radiotherapy [1]. According to their study, the maximum safe dose of ICD is uncertain; generally, 2 Gy is used as a reference [1]. Several studies have reported on the radiation dose needed to result in ICD damage [6–11]. A summary of *in vivo* studies of thoracic radiotherapy with ICD is presented in Table 1. In our patient, the mean and maximum ICD doses *in vivo* were 0.89 Gy and 2.23 Gy, respectively, and follow-up duration (> 36 months) was relatively longer than in prior *in vivo* studies.

**Table 1:** Summary of thoracic radiotherapy with implantable cardioverter-defibrillator *in vivo* studies

Study	No. of patients	RT dose/fraction (Gy)	Energy (MV)	ICD dose (maximum dose, Gy)	Used dosimeter	Follow-up duration (mo)	Outcome
Thomas *et al*. [6]	1	56/28	18	< 0.5	NR	> 1.6	Reset to fallback mode
Nemec *et al*. [7]	1	59.4/33	NR	NR	NR	NR	Runaway ICD, resulting in polyform VT, implement of cardiopulmonary resuscitation during RT
Zaremba *et al*.^a^ [8]	5	37	6/18	37	NR	2.5–13.4	Reset to backup mode (*n* = 1)
Ahmed *et al*. [9]	1	69.6/36	15	52.4	NR	6	No failure
Scobioala *et al*.^b^ [10]	1	25.2/14 (conformal RT) 35/7 (SBRT)	6/15 6	15.85	TLD	16	No failure
Hudson *et al*. [11]	2	70/32	6/18	6.8	TLD	NR	No failure
Our patient	1	60/30	10	2.23	MOSFET	> 36	No failure

*Abbreviations: ICD* Implantable cardioverter-defibrillator, *MOSFET* Metal oxide semiconductor field effect transistor, *NR* Not recorded, *RT* Radiotherapy, *SBRT* Stereotactic body radiotherapy, *TLD* Thermoluminescent detector, *VT* Ventricular tachycardia

^a^The study was performed in ICD-implanted pigs

^b^Treatment combined with conformal RT and SBRT

These prior reports, however, were mainly focused on devices, direct or scattered radiation, and electromagnetic noise. ICDs are composed of an ICD generator and of wires (electrode and lead). Electrode wires are connected to the device generator and passed through a vein to the right chambers of the heart. The lead usually lodges in the apex or septum of the right ventricle.

There is no safe threshold dose of electrode and lead. The leads are generally considered to be insensitive to radiation, but one case report claims irradiation-induced damage of the leads resulting in shock coil failure [2–4, 12]. We did not estimate the radiation dose of lead and electrode in the treatment room. Partial lead and electrode were included in the radiotherapy fields; therefore, predicting the CIED dose is possible only by DVH owing to increase in the uncertainty of measuring the actual dose at < 5 cm distance between the radiotherapy field and the device [13]. The expected mean and maximum doses of the lead and electrode on the DVH were 17.12 Gy and 55.72 Gy in the lead and 1.81 Gy and 7.10 Gy in the electrode, respectively. Kirova *et al.* investigated the correlation with the lead dose and failure of the pacemaker [14]. They delivered thoracic radiotherapy with 30 Gy in 10 fractions in which the lead dose was converted to 0.1–23 Gy approximately in a conventional radiotherapy dose. The patient had a good response of the tumor and no failure of the device. According to Hristova *et al.*, the failure of ICD did not occur after 68.1 Gy to the electrodes, and they commented that the dose to the electrodes was not associated with malfunction of the ICD [15]. Even though partial lead and electrode were located in the radiotherapy fields, any events related to ICD damage did not happen in our patient.

High photon energy of >10 MV makes it possible to produce neutrons, which affects the function of CIEDs, and Salerno *et al.* recommended the application of low energy of ≤6 MV [16]. Gelblum *et al*. suggested RT with energy < 10 MV [17], whereas Hashii *et al.* compared the ICD failures between 10 and 18 MV [18]. They found no failures at 10 MV but frequent failures at 18 MV. We used 10 MV of photon energy for a less hot dose area and more homogeneous dose distribution than 6 MV.

In addition to photon energy, dose rate has to be considered. According to Mouton *et al*., failure of CIEDs can occur with a high dose rate, and they observed high risk of failure at 8 Gy/min [19]. In our patient, radiation was delivered at the dose rate of 4 Gy/min, and no adverse events occurred.

Most of the studies of radiotherapy with CIEDs measured the dose of CIEDs using a thermoluminescent dosimeter, whereas we used MOSFET because of its linearity and sensitivity to very few radiation doses, as well as convenience of allowing an immediate reading and its low cost [10, 11, 13].

Despite > 2 Gy delivered to the ICD and a high dose of > 50 Gy to the lead, our patient with an ICD underwent radiotherapy successfully with complete remission of tumor and no complications. Our patient’s case shows that radiotherapy may be a safe and effective treatment modality through careful monitoring of ICDs in patients with lung cancer who have ICDs.

## Abbreviations

CCRT: Concurrent chemoradiotherapy
CIED: Cardiac implantable electronic device
DVH: Dose-volume histogram
ICD: Implantable cardioverter-defibrillator
MOSFET: Metal oxide semiconductor field effect transistor
RT: Radiotherapy
SBRT: Stereotactic body radiotherapy
TLD: Thermoluminescent detector
VT: Ventricular tachycardia
